# Iodide of Potassium

**Published:** 1862-07

**Authors:** 


*Iodide of Potassium.*—This salt is one of the remedies which has of late steadily increased in general favor. This may, perhaps, to some extent be explained by the fact to which we have frequently adverted, that recent researches have proved many hitherto obscure affections to be really due to remote syphilitic taint. Although very valuable in some forms of rheumatism, and in procuring the absorption of inflammatory products generally, yet it is against the tertiary forms of syphilis that this salt best shows its specific power. Of late years the customary dose has much increased, and eight to ten grains is now with many of our hospital surgeons an ordinary prescription. Considering the enormous quantities which are prescribed both in hospital and private practice, and the rarity of any observable ill effects, it is quite clear that for the most part it exerts no evil influence. Wasting of the breasts and testes, the bugbears of former times, are now never heard of, and anything like iodism is certainly exceptional. The addition of ammonia to all mixtures containing the iodide of potassium is now a very common practice. We mentioned it some years ago as having been strongly recommended, amongst others, by Dr. Gull at Guy's and by Mr. Paget at St. Bartholomew's. Mr. Paget used to remark that he considered five grains of the iodide with half a drachm of sal volatile equal to ten grains of the iodide alone. With some surgeons, and especially in the treatment of syphilitic diseases of the nerves or brain, the addition of tincture of nux vomica is frequently made.—*Med. Times and Gaz., Jan.* 4, 1862—*Am. Journal of Medical Sciences.*

---

A DECREE, creating two new chairs in the Faculty of Medicine of Paris, has been published in the *Moniteur*. The first, that of Comparative Medicine, has been given to M. Rayer, who has also been named Dean of the Faculty, in lieu of M. Dubois. The other, that of Histology, is given to M. Charles Robin.

